# Up‐regulation of circRNA_0068481 promotes right ventricular hypertrophy in PAH patients via regulating miR‐646/miR‐570/miR‐885

**DOI:** 10.1111/jcmm.16164

**Published:** 2021-03-12

**Authors:** Hong‐mei Guo, Zi‐peng Liu

**Affiliations:** ^1^ Ultrasonography Department Weinan Maternal and Child Health Hospital Weinan China; ^2^ UItrasonic Diagnosis Department Hanzhong Central Hospital Hanzhong China

**Keywords:** circRNA_0068481, EYA3, miRNA, Pulmonary arterial hypertension, Right ventricular hypertrophy

## Abstract

CircRNA‐0068481 and several miRNAs are important in the pathogenesis of right ventricular hypertrophy (VH), while the inhibition of eye absent transcriptional coactivator and phosphatase 3 (EYA3) was proved to reverse vascular remodelling in rats. In this study, we tried to study the diagnostic value and mechanistic role of circRNA_0068481 in the diagnosis of RVH in PAH patients. qPCR was done to measure circRNA‐0068481, miR‐646, miR‐750, miR‐885 and EYA3 mRNA expression. Luciferase assay was done to explore the regulatory relationship between circRNA‐0068481/EYA3 and the miRNAs. Western blot was done to measure EYA3 expression in AC16 cells. The expression of circRNA‐0068481, miR‐646 and miR‐570 showed a considerable capability to diagnose RVH in PAH patients. The luciferase activity of circRNA‐0068481 was remarkably suppressed by miR‐646, miR‐570 or miR‐885. The luciferase signal of EYA3 was also inhibited by miR‐646, miR‐570 and miR‐885. Up‐regulation of circRNA‐0068481 expression in AC16 significantly decreased miR‐646, miR‐570 and miR‐885 expression, and up‐regulated EYA3 expression, whereas circRNA‐0068481 down‐regulation significantly increased miR‐646, miR‐570 and miR‐885 expression, and repressed EYA3 expression. CircRNA_0068481 sponged several miRNAs including miR‐646, miR‐570 and miR‐885. These miRNAs were all found to target the expression of EYA3 mRNA, which is involved in the onset of right ventricular hypertrophy. Therefore, it can be concluded that the up‐regulation of circRNA_0068481 can predict the diagnosis of right ventricular hypertrophy in pulmonary arterial hypertension patients.

**Abbreviations**
6MWD: 6‐minute walking distance
BMI: body mass index;
CO: cardiac output;
HR: heart rate;
mPAP: mean pulmonary arterial pressure;
NT‐proBNP: N‐terminal prohormone brain natriuretic peptide;
PAH: pulmonary arterial hypertension
PCWP: pulmonary capillary wedge pressure;
PVR: pulmonary vascular resistance;
RAP: right atrial pressure;
RVEF: right ventricular ejection fraction;
VH: ventricular hypertrophy

## 1 INTRODUCTION

Pulmonary arterial hypertension (PAH) is a kind of disorder that impacts both the heart and pulmonary vasculature, triggering right heart failure (RHF), right ventricular hypertrophy, and dilatation.^1^ In fact, RHF can be life‐threatening in PAH patients.^2^ Diastolic dysfunction of the heart, the symptoms of which are identical to PAH, is featured by changed filling patterns, extended relaxation phase and intrinsic stiffness in diastolic movement. Several researches have displayed raised RAP in individuals with PAH.^3^ Some imaging studies also showed changed filling patterns featured by elevated atrium induced filling.^4^ On top of that, extended RV time has actually been observed in many PAH patients.^4^ Nonetheless, many measurements of diastolic functions are all strongly dependent on load. For that reason, it still remains uncertain whether PAH patients are suffering from real RV diastolic impairments.^5^, ^6^ Therefore, the existence of RV diastolic impairments in PAH patients was investigated based on the diastolic PV relationship established via several PV loops under various loading conditions. However, due to its cardiopulmonary compromise, the procedure is considered as too invasive.^6^

EYA3 is an orthologous gene previously shown to encode several proteins of transcriptional co‐activators, which are commonly co‐expressed along with Pax6, Six2, Six1 and Dach genes.^7^, ^8^, ^9^, ^10^ It has actually been assumed that the Eya genes are involved in eye development by interacting with Pax6 as well as Dach genes.^11^ Previous research has revealed that EYA‐PTP likewise enhances endothelial cell migration as well as angiogenesis, while the inhibition of EYA‐PTP activity can be anti‐angiogenic, but EYA3‐PTP down‐regulation plays no visible role in the function of lung endothelium in animal models.^12^, ^13^, ^14^ It is believed that the inhibition of VEGF causes the apoptosis of endothelial cells during diseases, whereas the compensatory reprogramming in the tyrosine kinase signalling pathways results in the development of apoptosis‐resistant cells.^15^, ^16^ It was also reported that the inhibition of EYA3 phosphorylation impairs DNA damage repairs.^17^

As circular RNAs (circRNAs) cannot be degraded easily by nucleases, they are good biomarkers for illness diagnosis.^18^ CircRNAs are associated with the progression and development of heart diseases.^19^ The silencing of circRNA_010567 up‐regulated miR‐141 expression while down‐regulating TGF‐1 to control the severity of myocardial fibrosis.^20^ A previous research found that circ_0068481 was dramatically up‐regulated in pulmonary hypertension patients.^21^

Circular RNAs such as circRNA_0068481 were demonstrated to be a diagnostic biomarker for various diseases.^22^ Similarly, several miRNAs are important in the onset of right ventricular hypertrophy, and the inhibition of EYA3 was proved to reverse vascular remodelling in rats.^17^, ^23^, ^24^ In this study, we tried to study the value of circRNA_0068481 in the diagnosis of RVH in PAH patients. By enrolling a group of PAH patients with or without RVH, we studied the role of certain genes potentially involved in the signalling pathway regulated by circRNA_0068481.

## 2 MATERIALS AND METHODS

### 2.1 Human samples

A total of 132 PAH patients were divided into 1. PAH (‐) RVH (‐) group (N = 52); 2. PAH (+) RVH (‐) group (N = 42); and 3. PAH (+) RVH (+) group (N = 38). We then collected and compared the basic characteristics of the patients, including their age, body mass index (BMI), sex, 6‐minute walking distance (6MWD), mean pulmonary arterial pressure (mPAP), cardiac output (CO), pulmonary vascular resistance (PVR), right ventricular ejection fraction (RVEF), pulmonary capillary wedge pressure (PCWP), right atrial pressure (RAP), heart rate (HR) and N‐terminal prohormone brain natriuretic peptide (NT‐proBNP), as presented in Table 1. The inter‐group comparison was done using one‐way ANOVA. Institutional ethical committee of Hanzhong Central Hospital has approved the protocol of this study. All methods were done in accordance with the last vision of the Declaration of Helsinki. Written informed consent was obtained from all patients or their first‐degree relatives before the study.

**Table 1:** Patient basic and clinical characteristics

Characteristics	PAH(‐)RVH(‐) (N = 52)	PAH(+)RVH(‐) (N = 42)	PAH(+)RVH(+) (N = 38)	*P* value
Age, years	54.3 ± 5.9	45.5 ± 4.8	46.1 ± 3.7	0.506
Sex, male	48/4	41/1	38/0	0.240
BMI (kg/m2)	24.5 ± 4.2	24.1 ± 4.2	24.5 ± 3.8	0.281
6MWD, m	493.5 ± 112.5	485.6 ± 78.5	478.6 ± 83.5	0.571
mPAP, mmHg	16.5 ± 2.2	48.5 ± 12.5	47.2 ± 18.5	0.624
CO, L/min	6.5 ± 1.1	5.4 ± 0.8	2.8 ± 0.4	0.526
PVR, dynes.S/cm5	125.5 ± 77.5	653.6 ± 226.7	663.1 ± 253.7	0.207
RVEF, %	58.1 ± 6.3	38.5 ± 4.2	25.5 ± 3.3	0.465
PCWP, mmHg	7.5 ± 2.5	8.2 ± 3.4	8.7 ± 3.8	0.543
RAP, mmHg	3.2 ± 2.1	7.4 ± 5.3	7.1 ± 3.8	0.524
HR, bpm	71.5 ± 7.2	88.3 ± 16.5	85.3 ± 17.1	0.879
NT‐proBNP, pg/L	138.5 ± 116.5	1457.5 ± 1834.5	1587.5 ± 1774.2	0.265

### 2.2 RNA isolation and real‐time PCR

The total RNA in each sample was separated by using a mirVana kit (Ambion, Hercules, CA). The quality and concentration of separated total RNA samples was evaluated by NanoDrop ND‐3000. The isolated total RNA, including circRNAs and miRNAs, in each sample was subject to reverse transcription done by utilizing a miRCURY qPCR assay kit (Exiqon, Vedbaek, Denmark) to generate corresponding cDNA templates. Then, to determine the relative expression of circRNA‐0068481 (Forward: 5’‐TATCTGCCCAAGGAGAGCAT‐3’; Reverse 5’‐TATTATCCATGGGAGGGAAGGT‐3’), miR‐646 (Forward: 5’‐AGCAGCTGCCTCTGAG‐3’; Reverse: 5’‐ GAACATGTCTGCGTATCTC‐3’), miR‐570 (Forward: 5’‐ GAAAACAGCAATTACCTTTG‐3’; Reverse: 5’‐ GAACATGTCTGCGTATCTC‐3’), miR‐885 (Forward: 5’‐ CCATTACACTACCCTGC‐3’; Reverse: 5’‐ GAACATGTCTGCGTATCTC‐3’) and EYA3 mRNA (Forward: 5’‐ GCAGTAGCCAGCATCTCAAACC‐3’; Reverse: 5’‐ GTCTGACCTGTGACTCCAAAGC‐3’) in each sample, qPCR was done with SYBR Green master mix in conjunction with specific primers and probes designed by Exiqon (Vedbaek, Denmark) on a 7500 qPCR machine (Applied Biosystems, Foster City, CA) to determine the relative expression of circRNA‐0068481, miR‐646, miR‐570, miR‐885 and EYA3 mRNA by utilizing the 2^‐ΔΔCT^ approach.^25^

### 2.3 Cell culture and transfection

AC16 cells were maintained in standard RPMI‐1640 added with 10% foetal bovine serum as well as 1% penicillin/streptomycin, at 37°C and 5% CO2. In this study, two cell models were used. In cell model I, there were two AC16 cell groups of 1. NC; 2. P‐circRNA_0068481 (AC16 cells transfected with P‐circRNA_0068481 plasmid). In cell model II, there were two AC16 cell groups of 1. NC siRNA; 2. CircRNA_0068481 siRNA. All transfection was done by utilizing Lipofectamine 3000 (Invitrogen, Carlsbad, CA) for 48 h.

### 2.4 Construct construction, mutagenesis and luciferase assay

Our binding site screening for miR‐646 indicated that miR‐646 could potentially bind to circRNA‐0068481. Then, the wild‐type circRNA‐0068481 luciferase vector was established by inserting the wild‐type sequence of circRNA‐0068481 containing the miR‐646 binding site into a pGL3 reporter vector (Promega, Madison, WI). A site‐directed mutation was induced in the miR‐646 binding site of circRNA‐0068481 sequence, which was then also inserted into a pGL3 reporter vector to establish the mutant‐type circRNA‐0068481 luciferase vector. Then, AC16 cells were transfected with miR‐646 and wild‐type/mutant‐type circRNA‐0068481 luciferase vector. Forty‐eight hours after transfection, the luciferase signal of the cells was analysed by making use of the Dual Luciferase reagent (Promega, Madison, WI) on a Luminoskan luminometer (Thermo lab systems, Franklin, MA). Similarly, miR‐570‐circRNA‐0068481, miR‐885‐circRNA‐0068481 and miR646/miR‐570/miR‐885**‐**EYA3 interaction was studied.

### 2.5 Western blot analysis

All samples were treated in a RIPA lysis buffer to collect total protein, which was then separated by 10% SDS‐PAGE gel and transferred onto a nitrocellulose membrane. After being probed sequentially with primary anti‐EYA3 antibodies (1:5000, ab95876, Abcam, Cambridge, MA) and HRP‐conjugated secondary antibodies (1:5000, ab6789, Abcam, Cambridge, MA), the protein blots were visualized by using a chemiluminescence reagent to quantify EYA3 expression with ImageJ v1.4.9 software.

### 2.6 Statistical analysis

Data were shown as Mean ± SD. Comparisons among multiple groups were done using one‐way ANOVA and Tukey's test as post hoc test in SPSS 19.0 (IBM, Chicago, IL) software. P values of ≤ 0.05 were taken into consideration as statistically significant.

## 3 RESULTS

### 3.1 Basic patient characteristics

We compared the basic and clinical characteristics among the three groups of 1. PAH(‐)RVH(‐) (N = 52); 2. PAH(+)RVH(‐) (N = 42); and 3. PAH(+)RVH(+) (N = 38), including their age, sex, BMI, 6MWD, mPAP, CO, PVR, RVEF, PCWP, RAP, HR and NT‐proBNP. We found no inter‐group difference in age, sex and BMI. Besides, no remarkable difference was found between PAH(‐)RVH(‐) and PAH(+)RVH(‐) patients in terms of 6MWD, CO and PCWP. However, the mPAP, PVR, RAP, HR and NT‐proBNP were elevated in PAH(+)RVH(‐) patients than in PAH(‐)RVH(‐) patients, whereas the RVEF was apparently lower in PAH(+)RVH(‐) patients when compared with PAH(‐)RVH(‐) patients. Moreover, we found no apparent difference between PAH(+)RVH(‐) and PAH(+)RVH(+) patients in terms of basic characteristics and most of the clinical characteristics except that CO and RVEF were obviously higher in PAH(+)RVH(‐) patients when compared with PAH(+)RVH(+) patients.

### 3.2 The expression of circRNA‐0068481, miR‐646 and miR‐570 showed a considerable capability to diagnose RVH in PAH patients

**FIGURE 1:**
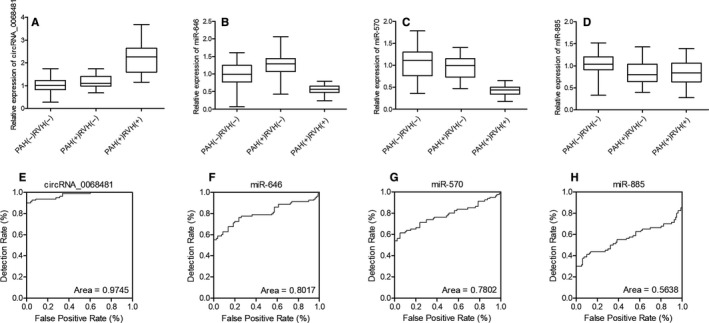
RVH was correlated with up‐regulation of circRNA‐0068481 and down‐regulation of miR‐646 and miR‐570. A, The expression of circRNA‐0068481 was elevated in PAH(+)RVH(+) patients when compared with PAH(‐)RVH(‐) and PAH(+)RVH(‐) patients. B, The expression of miR‐646 was decreased in PAH(+)RVH(+) patients when compared with PAH(‐)RVH(‐) and PAH(+)RVH(‐) patients. C, The expression of miR‐570 was decreased in PAH(+)RVH(+) patients when compared with PAH(‐)RVH(‐) and PAH(+)RVH(‐) patients. D, No obvious difference was observed for the expression of miR‐885 in PAH(+)RVH(+), PAH(‐)RVH(‐) and PAH(+)RVH(‐) patients. E, ROC analysis showed that the expression of circRNA‐0068481 was capable of predicting RVH in PAH patients. F, ROC analysis showed that the expression of miR‐646 was capable of predicting RVH in PAH patients. G, ROC analysis showed that the expression of miR‐570 was capable of predicting RVH in PAH patients. H, ROC analysis showed that the expression of 885 was not capable of predicting RVH in PAH patients

qPCR was done to analyse circRNA‐00684815, miR‐646, miR‐570 and miR‐885 expression in patients in the three groups. CircRNA‐00684815 expression was remarkably increased in PAH(+)RVH(+) patients when compared with PAH(‐)RVH(‐) and PAH(+)RVH(‐) patients (Figure 1A), but miR‐646 (Figure 1B) and miR‐570 (Figure 1C) expression was notably suppressed in PAH(+)RVH(+) patients when compared with PAH(‐)RVH(‐) and PAH(+)RVH(‐) patients. No obvious difference in miR‐885 expression was found among the PAH(+)RVH(+), PAH(‐)RVH(‐) and PAH(+)RVH(‐) patients (Figure 1D). Moreover, we did the ROC analysis to evaluate the value of circRNA‐0068481, miR‐646, miR‐570 and miR‐885 in predicting the RVH of PAH patients. The AUCs for circRNA‐0068481 (Figure 1E), miR‐646 (Figure 1F), miR‐570 (Figure 1G) and miR‐885 (Figure 1H) were 0.96, 0.82, 0.78 and 0.58, respectively. These results indicated that circRNA‐0068481, miR‐646 and miR‐570 were capable to diagnose the RVH of PAH patients.

### 3.3 Luciferase assays revealed a regulatory network of circRNA‐0068481, miR646/miR‐570/miR‐885 and EYA3

**FIGURE 2:**
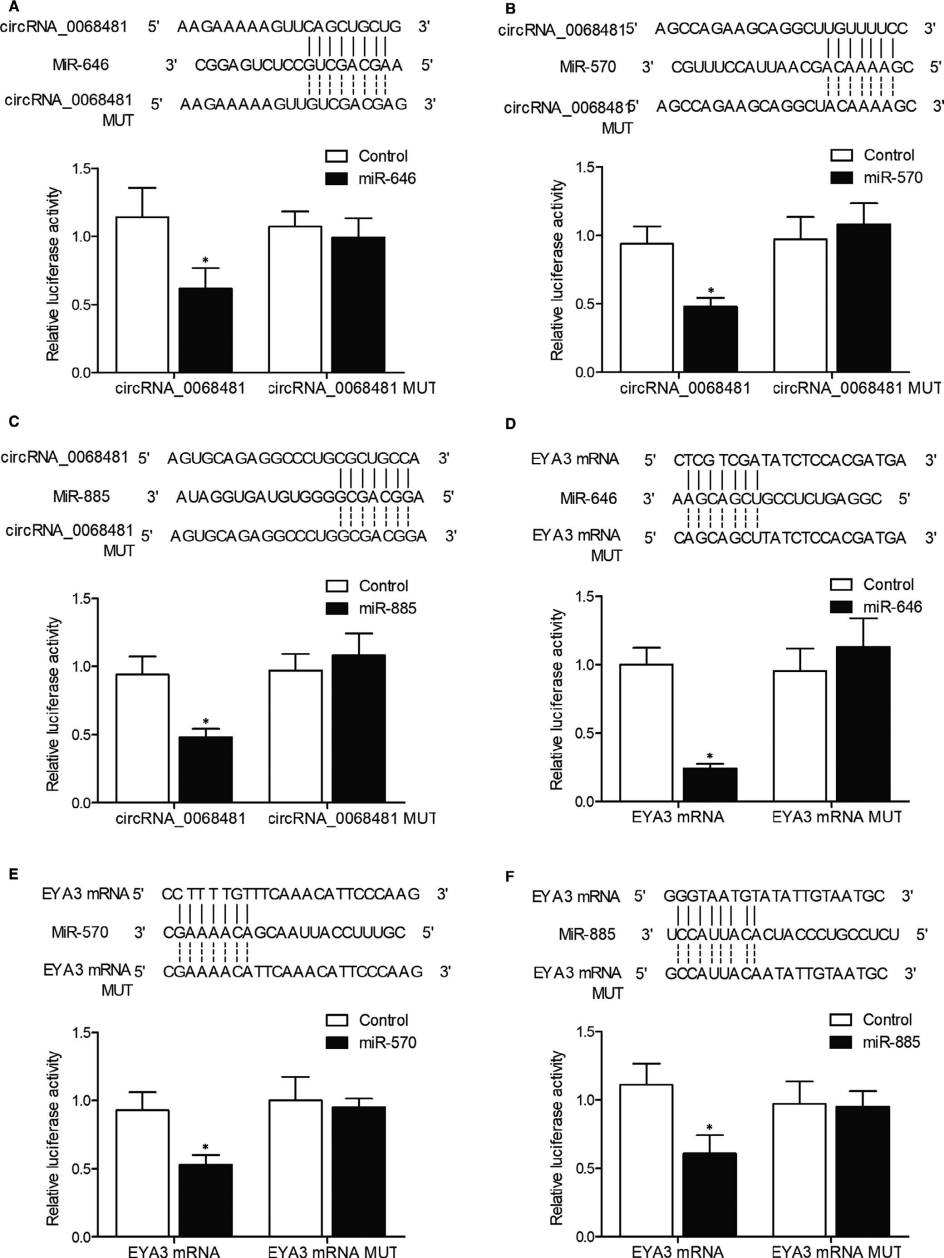
Luciferase assays revealed the regulatory relationships among circRNA_0068481/miR‐646, circRNA_0068481/miR‐570, circRNA_0068481/miR‐885, miR‐646/EYA3, miR‐570/EYA3 and miR‐885/EYA3 (MUT: mutant; *P value < 0.05 vs. control). A, Sequence analysis and luciferase assay indicated binding of miR‐646 to circRNA‐0068481. B, Sequence analysis and luciferase assay indicated binding of miR‐570 to circRNA‐0068481. C, Sequence analysis and luciferase assay indicated binding of miR‐885 to circRNA‐0068481. D, Sequence analysis and luciferase assay indicated binding of miR‐646 to EYA3. E, Sequence analysis and luciferase assay indicated binding of miR‐646 to EYA3. F, Sequence analysis and luciferase assay indicated binding of miR‐646 to EYA3

Binding site screening for miR‐646 indicated that miR‐646 could potentially bind to circRNA‐0068481. Mutant‐ and wild‐type circRNA‐0068481 luciferase vectors were established and transfected into AC16 cells with miR‐464. Wild‐type circRNA‐0068481 activity was effectively suppressed by miR‐646 in AC16 cells, whereas the mutant circRNA‐0068481 was not affected (Figure 2A). Binding site screening for miR‐570 indicated that miR‐570 could potentially bind to circRNA‐0068481. Mutant‐ and wild‐type circRNA‐0068481 luciferase vectors were established and transfected into AC16 cells with miR‐570. The luciferase activity of wild‐type circRNA‐0068481 was effectively suppressed by miR‐570 in AC16 cells, whereas the mutant circRNA‐0068481 was not affected (Figure 2B). Binding site screening for miR‐885 indicated that miR‐885 could potentially bind to circRNA‐0068481. Mutant‐ and wild‐type circRNA‐0068481 luciferase vectors were established and transfected into AC16 cells with miR‐885. The luciferase activity of wild‐type circRNA‐0068481 was effectively suppressed by miR‐885 in AC16 cells, whereas the mutant circRNA‐0068481 was not affected (Figure 2C). Binding site screening for miR‐646 indicated that miR‐646 could potentially bind to EYA3. Mutant‐ and wild‐type EYA3 luciferase vectors were established and transfected into AC16 cells with miR‐464. The luciferase activity of wild‐type EYA3 was effectively suppressed by miR‐464 in AC16 cells, whereas the mutant EYA3 was not affected (Figure 2D). Binding site screening for miR‐570 indicated that miR‐570 could potentially bind to EYA3. Mutant‐ and wild‐type EYA3 luciferase vectors were established and transfected into AC16 cells with miR‐570. The luciferase activity of wild‐type EYA3 was effectively suppressed by miR‐570 in AC16 cells, whereas the mutant EYA3 was not affected (Figure 2E). Binding site screening for miR‐885 indicated that miR‐885 could potentially bind to EYA3. Mutant‐ and wild‐type EYA3 luciferase vectors were established and transfected into AC16 cells with miR‐885. The luciferase activity of wild‐type EYA3 was effectively suppressed by miR‐885 in AC16 cells, whereas the mutant EYA3 was not affected (Figure 2F).

### 3.4 Alteration of circRNA‐0068481 effectively changed the expression of miR‐646, miR‐570, miR‐885 and EYA3

**FIGURE 3:**
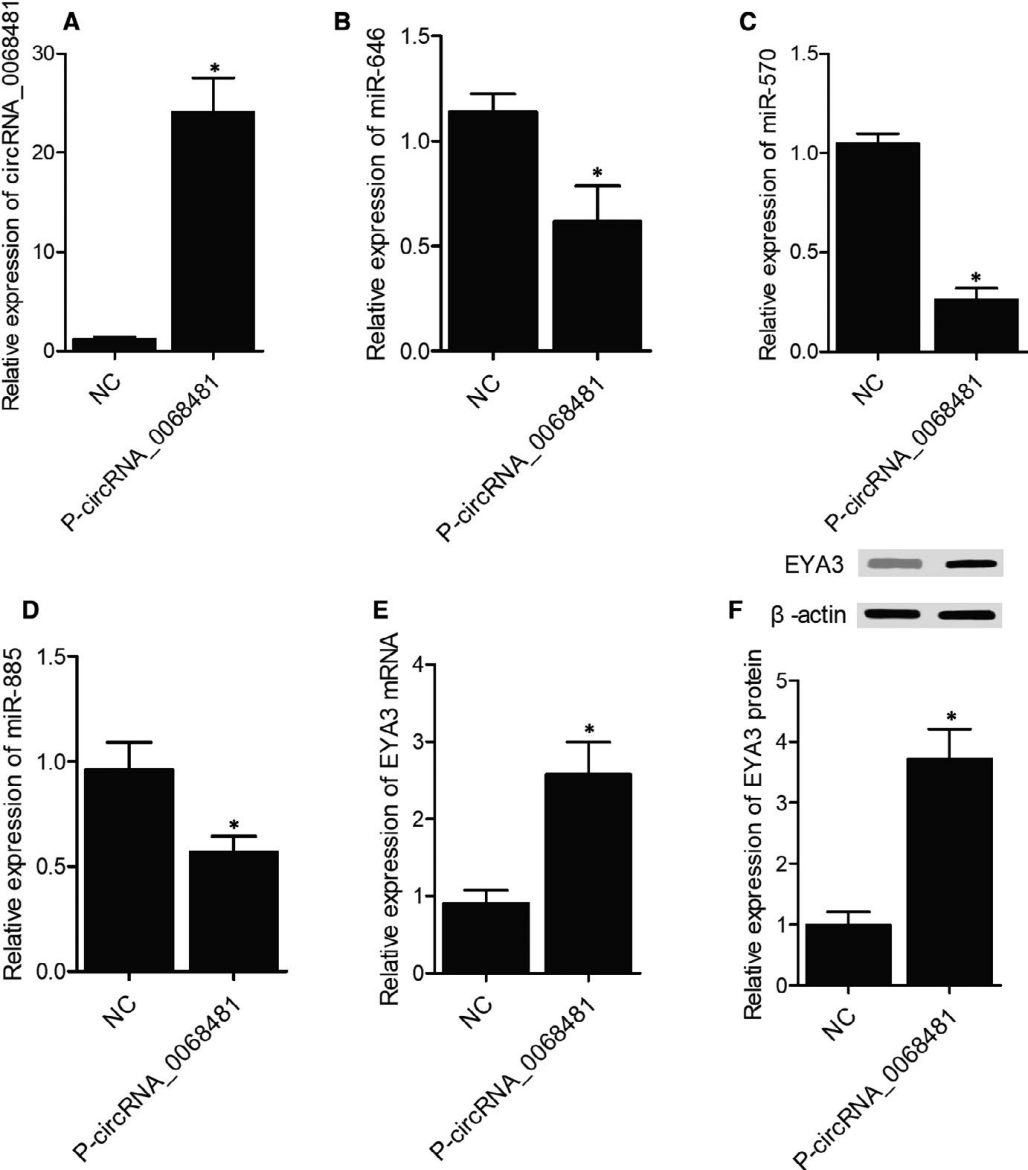
Overexpression of circRNA‐0068481 suppressed the expression of miR‐646/miR‐570/miR‐885 and up‐regulated the expression of EYA3 in AC16 cells (NC: negative control; * P value < 0.05 vs. NC). A, The expression of circRNA‐0068481 was dramatically enhanced by p‐circRNA‐0068481 in AC16 cells. B, The expression of miR‐646 was remarkably repressed by p‐circRNA‐0068481 in AC16 cells. C, The expression of miR‐570 was remarkably repressed by p‐circRNA‐0068481 in AC16 cells. D, The expression of miR‐885 was remarkably repressed by p‐circRNA‐0068481 in AC16 cells. E, The expression of EYA3 mRNA was remarkably up‐regulated by p‐circRNA‐0068481 in AC16 cells. F, The expression of EYA3 protein was remarkably up‐regulated by p‐circRNA‐0068481 in AC16 cells

**FIGURE 4:**
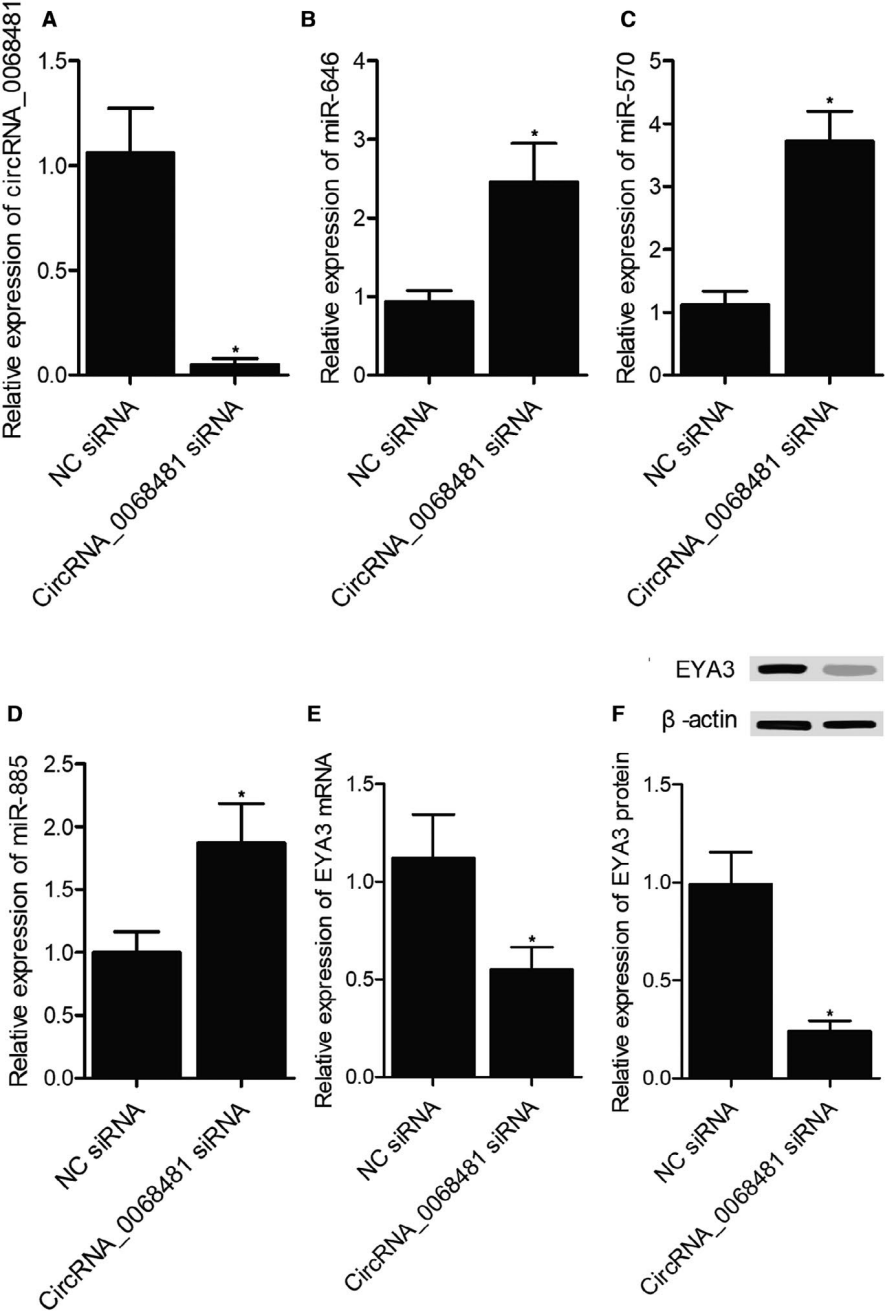
Suppression of circRNA‐0068481 expression up‐regulated the expression of miR‐646/miR‐570/miR‐885 and inhibited the expression of EYA3 in AC16 cells (NC siRNA: negative control siRNA; *P value < 0.05 vs. NC siRNA). A, The expression of circRNA‐0068481 was dramatically suppressed by circRNA‐0068481 siRNA in AC16 cells. B, The expression of miR‐646 was remarkably up‐regulated by circRNA‐0068481 siRNA in AC16 cells. C, The expression of miR‐570 was remarkably up‐regulated by circRNA‐0068481 siRNA in AC16 cells. D, The expression of miR‐885 was remarkably up‐regulated by circRNA‐0068481 siRNA in AC16 cells. E, The expression of EYA3 mRNA was remarkably inhibited by circRNA‐0068481 siRNA in AC16 cells. F, The expression of EYA3 protein was remarkably inhibited by circRNA‐0068481 siRNA in AC16 cells

We further overexpressed cricRNA‐0068481 in AC16 cells by transfecting p‐circRNA‐0068481 (Figure 3A). Accordingly, the expression of miR‐646 (Figure 3B), miR‐570 (Figure 3C) and miR‐885 (Figure 3D) was remarkably suppressed in AC16 cells by p‐circRNA‐0068481, whereas the expression of EYA3 mRNA (Figure 3E) and protein (Figure 3F) was notably increased in AC16 cells. Meanwhile, the expression of circRNA‐0068481 was repressed in AC16 cells by cricRNA‐0068481 siRNA (Figure 4A). As a result, the expression of miR‐646 (Figure 4B), miR‐570 (Figure 4C) and miR‐885 (Figure 4D) was remarkably increased in AC16 cells by circRNA‐0068481 siRNA. The expression of EYA3 mRNA (Figure 4E) and protein (Figure 4F) was significantly suppressed in AC16 cells by circRNA‐0068481 siRNA.

## 4 DISCUSSION

PAH can lead to right ventricular (RV) disorder, a death leading morbidity.^26^, ^27^ The prognosis of PAH is highly associated with certain RV parameters, including right atrial pressure and cardiac index.^5^, ^28^, ^29^ Wilkins et al found that sildenafil treatment reduced RV mass.^30^ Galie et al revealed that bosentan therapy was linked to enhancement in RV systolic functions.^31^ In this study, we recruited PAH patients with or without RVH to explore the mechanistic role of circRNA‐0068481. We found that RVH was significantly related with increased circRNA‐0068481 expression and decreased miR‐646 and miR‐570 expression, which were proved to act as potential biomarkers for RVH diagnosis.

EYA3 plays a number of biochemical roles.^32^, ^33^ Specifically, the tyrosine phosphatase activities of EYA3 have been linked to the progression as well as upkeep of lung vascular remodelling. It was presented that EYA3‐PTP enhances the survival of pulmonary vascular cells upon DNA damages, and the lungs of PAH patients showed much higher levels of genomic instability, DNA damages, and modified DNA repair mechanism.^34^, ^35^ It was also shown using proliferation and cells cycle assays that the co‐expression of c‐Src and Y527F in HEK293T cells caused enhanced cell proliferation. The same effect was additionally highlighted using flow cytometry analyses.^36^ It was thought that the functionality of EYA3 is to preserve the state of neural precursor in early development. As a consequence, neural progenitor cells deficient of EYA3 could not differentiate and hence underwent apoptosis.^37^

It was shown that serum circ_0068481 expression was dramatically higher in individuals with IPAH, indicating that circ_0068481 can predict IPAH. Furthermore, it was discovered that circ_0068481 expression was considerably higher in IPAH patients free of RHF, indicating that the expression of circ_0068481 was actually induced via IPAH.^22^ MiR‐646 is widely present in vascular endothelial cells and has been illustrated to play a vital role in angiogenesis.^38^ Li et al showed that miR‐646 is expressed in both normal cells and renal cell carcinoma, and promotes the angiogenesis of renal cells.^39^ The malignant progression in certain cancers such as endometrial cancer shows dysregulated miR‐646.^39^ Additionally, it was revealed that down‐regulated miR‐646 aids pancreatic cancer tumorigenesis.^40^ MiR‐570 is the most essential miRNA involved in the onset of alcoholic liver disease, which can advance to hepatocellular carcinoma.^41^ EdU staining and MTT assays revealed that hsa‐miR‐570‐3p mimics can substantially hinder the proliferation of TNBC cells.^42^ On top of that, hsa‐miR‐570‐3p expression is reduced in metastatic osteosarcoma compared with non‐metastatic osteosarcoma, indicating that miR‐570‐3p down‐regulation may enhance tumour metastasis.^42^, ^43^

From the results of high‐throughput miRNA sequencing, miRNA‑885‑3p was recognized as one of several miRNAs whose expression was down‐regulated in SPC‐A1/DTX cells resistant to the docetaxel treatment.^44^, ^45^, ^46^ MiR‐885‐5p up‐regulation was reported in pancreatic cancer and breast cancer.^47^ In breast cancer, the expression of E2F transcription factor 1 was down‐regulated by miR‐885, thus regulating MCF‐7 cell proliferation.^47^ A previous study has illustrated that miR‐885‐5p might be up‐regulated in osteosarcoma cells.^48^ In this study, we altered the expression of circRNA‐0068481 by overexpression and siRNA inhibition. The alteration of circRNA‐0068481 remarkably changed the expression of miR‐646, miR‐570, miR‐885 and EYA3.

## 5 CONCLUSION

In this study, we found that the signalling pathway regulated by circRNA_0068481 is involved in the pathogenesis of right ventricular hypertrophy. And thus, the up‐regulation of circRNA_0068481 can be used as a potential biomarker for the diagnosis of right ventricular hypertrophy in patients with pulmonary arterial hypertension.

## CONFLICT OF INTEREST

None.

## AUTHOR CONTRIBUTIONS

GHM and LZP planned the study, collected and analysed the data, and finished the manuscript. LZP collected the funding and literature, and read and approved the final manuscript.

## Data Availability

The datasets used and/or analysed during the current study are available from the corresponding author upon reasonable request.
